# Alternative Splicing in Myeloid Malignancies

**DOI:** 10.3390/biomedicines9121844

**Published:** 2021-12-06

**Authors:** Carmelo Gurnari, Simona Pagliuca, Valeria Visconte

**Affiliations:** 1 Department of Translational Hematology and Oncology Research, Taussig Cancer Institute, Cleveland Clinic, Cleveland, OH 44195, USA; gurnarc@ccf.org (C.G.); smnpag@gmail.com (S.P.); 2 Department of Biomedicine and Prevention, University of Rome Tor Vergata, 00133 Rome, Italy

**Keywords:** RNA-splicing, cancer, therapies

## Abstract

Alternative RNA splicing (AS) is an essential physiologic function that diversifies the human proteome. AS also has a crucial role during cellular development. In fact, perturbations in RNA-splicing have been implicated in the development of several cancers, including myeloid malignancies. Splicing dysfunction can be independent of genetic lesions or appear as a direct consequence of mutations in components of the RNA-splicing machinery, such as in the case of mutations occurring in splicing factor genes (i.e., *SF3B1*, *SRSF2*, *U2AF1*) and their regulators. In addition, cancer cells exhibit marked gene expression alterations, including different usage of AS isoforms, possibly causing tissue-specific effects and perturbations of downstream pathways. This review summarizes several modalities leading to splicing diversity in myeloid malignancies.

## 1. Introduction

**Figure 1:**
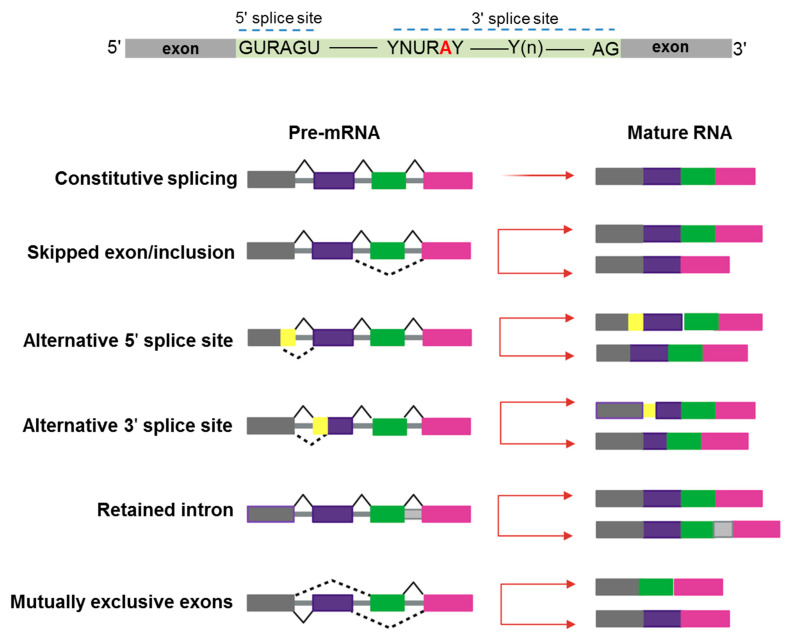
Schematic representation of constitutive and alternative splicing. Upper panel: 5′ and 3′ splice-site and branch-site consensus sequences are shown for mammalian U2-dependent introns. R indicates a purine, Y a pyrimidine, and N any other nucleotide. The branch point adenosine is indicated in red color.A tract of 10 to 20-nucleotides forms a pyrimidine tract (Yn) between the branch site and 3′ splice site. Modes of action of RNA-splicing from pre-messenger RNA to mature RNA are presented. Canonical transcripts are produced via constitutive splicing, while variability is naturally produced through different modes of alternative splicing.

In the last 10–15 years, advances in genomic technologies and deep RNA-sequencing in combination with whole-genome sequencing have enormously increased the chance to identify a global dysregulation in RNA-splicing and its association with gene mutations in hematologic malignancies [1]. Pre-mRNA splicing is a precise process in which non-coding sequences of a gene are excised and coding regions are joined together to form a mature RNA for subsequent protein translation [2]. Alternative splicing (AS) is a natural process creating transcript variability, and it is common to 95% of human genes. Splicing diversity can either be physiologic and present in normal individuals without leading to pathologic conditions or be potentially selective to induce human diseases, often resulting in the production of cancer vulnerability due to abnormal AS phenomena [3]. Indeed, aberrant AS produced at a single cell level can impair the subsequent cell development if occurring in key transcriptional factors crucial to assign cell identity [4,5]. Moreover, a variety of chemotherapeutic drugs can induce changes in AS modalities, typically intron retention, which is similar among different pharmacologic agents and produces a down-regulation of gene expression and phosphorylation [6]. Several modes of AS are represented in Figure 1.

The regulation of splicing is carried out by an orchestra of genes and proteins. Dysfunctions in the RNA-splicing machinery either due to molecular alterations or general perturbations of RNA-splicing regulation have been found in several human diseases [7]. Although splicing regulation has been less studied in normal development, alterations in RNA-splicing occur at different cellular levels also in this context. It is conceivable that RNA-splicing will be a pivotal mechanism in cellular fate, considering its role in mediating expression (on/off genes switching) and the finely tuned processes underpinning cellular hematopoietic development [8]. This also includes the activation and regulation of lineage transcriptional factors and master transcriptional factors dictating the fate of cells in the body and their functions. For instance, it has been reported that *RUNX1* gene is subjected to AS generating an ortholog product in hematopoietic stem cells (HSCs) [9], and *RUNX1* chromosomal translocation [t(12;21)] and the *RUNX1* mutations lead to specific acute myeloid leukemia (AML) subsets.

Given the occurrence of mutations in components of the RNA-splicing machinery in hematologic disorders, especially myelodysplastic syndromes (MDS) and AML, which are clonal disorders arising from genetic alterations of HSCs, a specific focus of the research community has been given to splicing abnormalities in normal hematopoiesis [10]. Transcriptomic studies have offered tools to compare the profile of genes of interest between abnormal and normal HSCs by revealing multiple splicing changes in HSC genes. In fact, several studies conducted in animal models have investigated the whole transcriptome of HSCs to understand how distinct genes (often associated with leukemia) might be regulated by AS in normal conditions. This has been the case of changes in splicing profiles by minor intron retention (*HoxA9)*, exon skipping (*Meis1*), and abundance of non-canonical isoforms (*Prdm16*) [11]. Similarly, intron retention and non-sense mediated decay (NMD) have been found at high proportions in HSCs during cell-specific activation [12]. Deep RNA-sequencing of early HSCs (CD34+ CD38− CD90+ CD45RA−) and CD34+ CD38+ progenitors demonstrated the presence of isoforms involved in HSC stemness. For example, different isoforms of *HMGA2* (long and short) have diverse distributions according to tissue specificity. Furthermore, regulation of isoform switch was attributed to SRSF1 and protein kinase CLK3 phosphorylation [13]. This pointed out that changes in RNA-splicing might be the molecular underpinnings of the mechanisms regulating the functions of HSCs. Similarly, RNA-binding proteins (RBPs) have been studied in regards to their involvement in RNA-splicing regulation, among other functions that they have in hematopoietic and non-hematopoietic tissues [14]. Indeed, AS and differential usage of splicing isoforms are regulated by post-translational modifications of RBPs, and several RNA-binding domains have been identified. However, the exact functional consequences of many of their interactions with RBPs remain to be fully elucidated [15]. The presence of regulatory cis-elements bound by RBPs located within 5′ or 3′ untranslated regions (UTRs) seems likely to increase transcriptome diversity [16]. A seminal study by Yu and colleagues demonstrated the role of BCAS2 (breast carcinoma amplified sequence 2), a component of the spliceosome in hematopoietic development. In particular, the authors generated a *Bcas2* knockout zebrafish model (*Bcas2*^−/−^ zebrafish), which showed severe impairment of HSCs and progenitor cells during hematopoiesis and found that p53-mediated apoptosis was associated with abnormal AS of Mdm4 [17].

## 2. De-Regulation of RNA-Splicing in Myeloid Malignancies

As aforementioned, RNA-splicing is significantly altered in myeloid malignancies. The involvement of altered RNA-splicing in myeloid disorders stems from the presence of tumor suppressors and/ or proto-oncogenes often characterized by AS producing isoforms of different lengths. Indeed, a large number of pivotal genes in cancer are characterized by disease-related AS, and the prototypic example is the generation of Bcl-XL or -XS proteins. In fact, intron 2 of Bcl-XL can be spliced in two different 5′ splice sites (ss). Similarly, the inclusion or exclusion of exon 9 will dictate the two isoforms of caspase 2 (2L and S) [18]. Indeed, according to the different retained/removed regions, isoforms with different lengths can be produced. For instance, Telomerase Reverse Transcriptase (*TERT*), a ribonucleoprotein polymerase consisting of reverse transcriptase activity maintaining telomere ends by addition of the telomere repeat TTAGGG, is characterized by a considerable number of alternative 5′ ss, exon skipping etc. [19]. Another example is *TP53,* with the majority of the AS characterized by intron retention and alternative 5′ ss [20].

Several studies have shown a global aberrant RNA-splicing in patients with MDS and AML compared to healthy individuals [21]. Initially, these studies were conducted with expressed sequence tags (ESTs), but the possibility of using only a few ESTs became soon a limitation of this technique [22]. In one study applying gene chip human transcriptome arrays (Affymetrix HTA 2.0) with 6 million different probes, about 78% of genes were found expressed in bone marrow mononuclear cells from MDS patients with the criterion that at least one aberrant AS had to be present compared to healthy individuals. About 27% of the genes were aberrantly spliced (86% in coding and 14% in non-coding regions). Soon after, deep-RNA sequencing was implemented. The use of this new technique allowed the association of abnormalities in AS with poor outcomes and the rate of AS with the presence of *U2AF1* mutations. A LASSO-Cox regression model was then used to predict the overall survival from the estimation of AS changes, finding a signature of affected genes spanning different pathways [23].

Pellagatti and colleagues applied RNA-sequencing to enriched CD34+ cells of 84 patients with MDS and healthy individuals. Patients carrying the most common splicing factor (SF) mutations (i.e., *SF3B1*, *SRSF2*, and *U2AF1*) showed convergent pathways. Significant abnormal AS events (intron retention, skipping exons, alternative 3′ ss) were identified across patients harboring SF mutations. Among several target genes, two genes involved in the regulation of cellular mitosis (*SEPT2* and *AKAP8*) were identified as common targets of *SF3B1* and *SRSF2* mutations after confirmation by knockdown experiments. Furthermore, genes associated with R-loop formation (*SETX* and *ATR*) were affected in both *SF3B1* and *SRSF2* mutant patients [24]. Another seminal paper was published by Shiozawa et al. [25] on RNA-sequencing analysis of 265 specimens of MDS, with a subsequent validation using CRISPR/Cas9-mediated gene editing. *SF3B1* mutations were associated with novel splicing mechanisms and abnormal 3′ ss of genes related to heme biosynthesis and iron metabolism (*ABCB7*, *PPOX*, *TMEM14C*) and cancer-specific genes (*NF1*, *PDS5A*, *DICER1*, and *PML)* [25]. These studies identified also other genes involved in iron trafficking and metabolism to be dysregulated in MDS. For instance, SLC25A37, encoding for an iron importer, was found abnormally spliced in *SF3B1* mutant MDS [26,27]. Conte and colleagues reported on the AS of hemoglobin genes in *SF3B1* mutant MDS, finding clues on the erythropoietin axis impairment typical of these patients, characterized indeed by various degrees of anemia [28]. Another gene, *EZH2*, was recognized as a common target associated with *SRSF2* and *U2AF1*^S34^ mutations. In particular, an exon skipping event was specifically related to *SRSF2* mutations. Previous studies have identified the inclusion of a poisoning exon in *EZH2* gene promoted by *SRSF2* mutations through the binding to a C-rich splicing enhancer subsequently degraded through NMD [29,30].

In regards to *U2AF1*, a combination of primary cell data and functional experiments showed that its mutations induce differential splicing of many genes affecting a variety of pathways, including DNA methylation (*DNMT3B*), X chromosome inactivation (*H2AFY*), DNA damage response, and apoptosis genes (*ATR*, *FANCA*, *CASP8)* by altering the preferentially 3′ ss motifs [31]. Later on, it was demonstrated that the introduction of isoform 1.1 in *H2AFY* (H2A Histone Family, Member Y) and the overexpression of the long isoform of *STRAP* (Serine/Threonine Kinase Receptor Associated Protein) were able to dictate cell fate and induce a phenotype resembling the one associated with S34 mutation in hematopoietic cells [32]. Very recently, it has been recognized that the phenomenon of AS might go beyond the mere presence of SF mutations, being, instead, a much broader aspect of the disease pathobiology. Indeed, AS of *DHX34* (DExH-Box Helicase 34) is the mechanism at the basis of the reduced expression of transcripts consisting in full-length coding regions. This reduced expression causes also a cascade of transcriptomic changes in other genes involved in NMD. The originality of this study resides in the identification of splicing models (set of exons and introns that can be spliced together), possibly producing phenocopies of somatic mutations. Moreover, the authors further speculated on the possibility of an upstream trans-acting factor common to more than one pathway [33].

## 3. Alternative Splicing as a Novel Mechanism of Neo-Antigens Production

Perturbations in RNA-splicing due to mutations in SF genes constitute also a modality that generates tumor neo-antigens. These neo-antigens can be private (specific to single patients), public (shared across patients) and disease-specific (not present in normal tissues of normal counterparts). Cancer-specific AS has been studied across large datasets of cancer types with the purpose of identifying shared novel exon-junctions. However, many novel exon-junctions are still biologically and functionally unexplored [34]. In-depth integrated analyses of RNA and whole-exome sequencing of tumors from 8705 donors, including 670 matched normal samples (TCGA and GTEx databases) from a variety of cancer types have shown that mutations in certain SFs (for instance, *SF3B1*, *U2AF1*) might generate immunogenic neo-antigens [35,36]. In several cases, mutations in SF genes led to abnormal open reading frames, retention of intronic sequences and in-frame insertions of codons, possibly forming neo-epitopes. Indeed, neo-junction-derived peptides were identified as putative neo-antigens in disease samples and verified based on bioinformatics prediction tools and mass-spectrometry. These studies, together with others [37,38], pointed out not only the frequency and abundance of novel splicing events but also the lack of recurrence and restriction of neo-antigens due to the high diversity in HLA haplotypes. Furthermore, neo-antigens may also be the product of errors in mis-splicing of exons and transcription of microsatellites, specifically indels, which, in normal conditions, are eliminated by NMD and not presented to the immune system while in cancer cells are found on the surface of antigen-presenting cells (APC) linked to MCH class I and II molecules [39].

**Figure 2:**
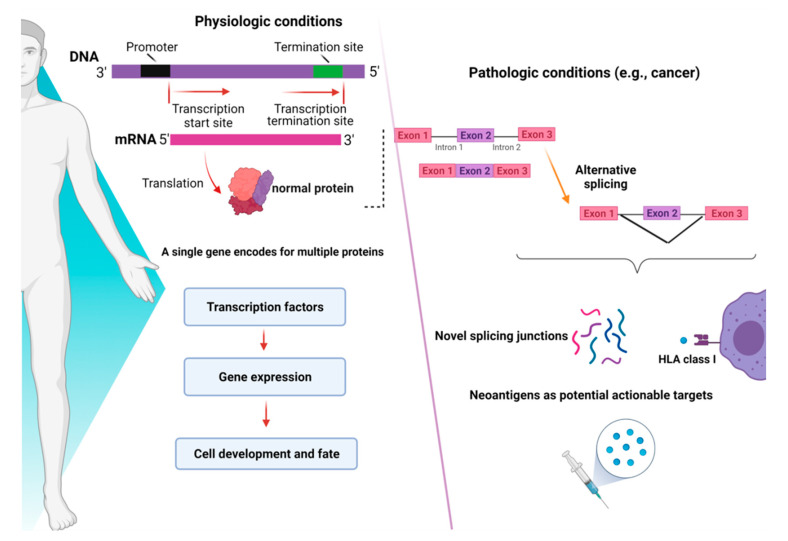
Consequences of alternative splicing in physiologic and pathologic conditions. In physiologic conditions (**left panel**), alternative splicing serves as a modulator of gene expression and regulates the function of transcriptional factors, ultimately dictating cell development and fate. In pathologic conditions (**right panel**), alternative splicing can produce novel junctions that, when exclusive to tumor cells, might represent actionable targets for immunotherapy. Images were generated (in part) using BioRender.

RNA-sequencing studies conducted in myeloid malignancies (MDS and AML) have shown that AS can generate novel protein junctions also through gain and loss of coding RNA sequences or by the production of frameshifts. These novel coding regions, when identified in patient samples, might potentially help the final diagnosis of some myeloid disorders, such as MDS, whose diagnostic criteria are sometimes relatively subjective (e.g., dysplasia assessment) [40]. In this study, specific disease-splicing events were classified as being selected for patients and not present in control samples. By testing for binding to class I MHC, 925 disease-specific splicing events were found. About 2% of these could be related to patients with one or more SF mutation, and some were consistently found in *SF3B1* (*FAM143A*, *FMNL1*, *PILRB*) and *SRSF2* (*CD2BP2*, *RASGRP2*) mutant cases. Studies conducted in induced pluripotent stem cells generated from MDS patient cells showed that *SF3B1* mutations can produce HLA-presented neo-antigens, opening a new avenue for targeted immunotherapy. Interestingly, using HLA-binding algorithms (netMHCpan 4.0), peptides derived from mutated transcripts of *SF3B1* showed a strong affinity for some class I HLA molecules (i.e., HLA-B*40:01), and their processing, presentation, and immunogenicity were functionally proven in vitro [41]. In particular, it has been demonstrated that certain cancers tend to present more neojunctions than others, irrespective of the observed mutational burden [36]. As discussed before, this is also true for MDS and AML, which present a high percentage of neosplicing events irrespective of SF mutations [40]. Besides mutation-produced neo-antigens, noncoding transcripts have also an immunogenic potential. Indeed, a combination of liquid chromatography tandem-mass spectrometry and RNA-sequencing has been used to detect noncoding transcripts, which can be created by introns, untranslated regions, and noncoding exons [42]. Splice-site-creating mutations are, in fact, predicted to generate an average of 2–2.5 neo-antigens per mutation [43]. In recent years, several workflows have been tested for the classification of these neo-antigens [44], and the optimization of bioinformatic tools has been growing extensively. By using RNA-sequencing for personalized neo-antigen prediction, a seminal study from Schischlik et al. [45] explored the neo-antigen repertoire in myeloid malignancies to find clues as to new targets for immunotherapy-based treatments. The neo-antigens repertoire was analyzed according to the presence and types of mutations, indels, fusion genes, and RNA-splicing errors. The majority of the mutations were of frameshift origin and caused the production of novel sequences, which were then analyzed for their binding affinity to MHC classes I and II. It is, thus, understandable how consequences of aberrant splicing, together with neo-antigens derived from mutated oncogenic proteins, in cancer cells may have a direct impact on the shape of the immune-peptidome presented to T cell effectors, finally contributing to mechanisms of immunoediting and clonal selection [46]. A summary of the role of mechanisms of AS in physiologic and pathologic conditions is presented in Figure 2.

## 4. RNA-Splicing-Based Targeted Therapies

Given the importance of RNA-splicing as a basic cellular process, therapeutic targeting, while being an attractive avenue, has also called for some cautions as minimal changes in splicing could influence equally normal cells (e.g., narrow therapeutic window). However, the knowledge of specific tumor-associated splicing events has continuously animated the research on therapeutic targeting of splicing. Below are described some RNA-splicing-based therapeutic approaches.

### 4.1. Antisense Oligonucleotides (ASOs)

ASOs are oligonucleotides of 15–25 bases in length and are reverse complement sequences of a specific RNA transcript region [47]. ASOs bind target RNA and preclude the direct access of SFs to the RNA target sequence or block/ silence enhancer elements of SFs, ultimately causing SFs to use alternative and proximal sequences. Several ASOs have been synthetically constructed to create loops to specifically block SFs or to target only mutant alleles in order to enhance selectivity and prevent alteration of normal sequences. Some studies have shown increased stability and simple internalization [1]. More recently, ASOs have also been shown to block the abnormal inclusion of a “poison exon” of BRD9 in *SF3B1* mutant cells [48].

**Figure 3:**
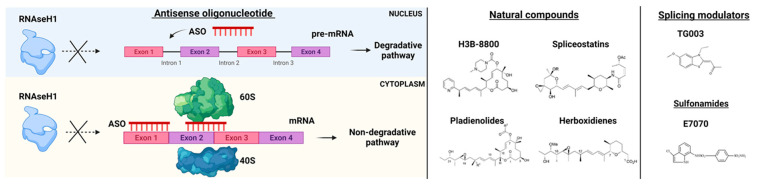
RNA-splicing-based targeted therapies. On the left, Antisense oligonucleotide (ASO) mechanisms of action. ASOs interfere with pre-mRNA by preventing RNaseH1 functions and blocking the interaction of mRNA with ribosomes (subunits 60S and 40S). On the right, examples of natural compounds, splicing modulators and sulfonamides used in pre-clinical and clinical models. Parts of the images were generated with BioRender.

Efforts in drug development have been made to generate ASOs inducing RNase H1-mediated cleavage of target RNAs. This is the case of the gapmer ASOs composed of an oligodeoxynucleotide portion flanked by 2′ modified nucleotides at both ends. RNase H1-dependent ASOs are able to reduce levels of both cytoplasmic mRNA and retained nuclear RNA. The differences in ASOs activity in both nuclear and cytoplasmic compartments are due to many factors, including not only the presence of RNAseH1 but also specific RNA sequences and structure, RNA stability, and levels of ASOs (Figure 3) [49].

Next-generation ASOs have hybrid structure antisense base-pairing sequences, which are linked to canonical binding sites of an SF. Further, ASOs have been constructed to induce usage of a cryptic ss or exon skipping. Novel methodologies incorporate ASOs in trans-splicing technology or Spliceosomal-Mediated RNA trans-splicing (SMaRT), which replaces the entire coding sequence of a target ss through ASO-containing plasmids targeting an endogenous intron of the mutant ss. This method has been used to correct mutations occurring in the 5′ ss or 3′ ss [47]. For instance, this approach has been applied to replace the first exon of the *HBB* (β-globin) gene in β-thalassemia [50]. Splicing restoration has also been tested using different versions of the spliceosomal snRNAs designed to base-pairing in a mutant 5′ ss. Furthermore, the introduction of genome editing technologies, including transcription activator-like nucleases (TALENs) and CRISPR/Cas9, have been implemented to correct splicing defects [51]. In fact, CRISPR/Cas9 was used to edit protein domains and identify drug targets by screening 192 chromatin regulatory domains in MLL-AF9/*Nras*^G12D^ models of AML [52]. Other groups applied a CRISPR/Cas9 method to edit multiple genes mutated in AML and track clonal dynamics in murine systems using Lin–Sca1+c-Kit+ (LSK) cells [53].

### 4.2. Small Molecules Agents

Small molecules have been tested in several models of spliceosome dysfunction. Specifically, H3B-8800, a selective and orally bioavailable modulator of normal and mutant SF3b complex, has shown dose-dependent modulation of splicing in pre-clinical xenograft models [54], but unfortunately, these data have not been confirmed by the phase I, first-in-human open-label, multicenter trial for patients with previously treated MDS, AML, and CMML (NCT02841540) [55]. The discovery of H3B-8800 follows a path of research efforts on natural products (Figure 3). Indeed, products derived from bacteria have been shown to bind the SF3b complex and disrupt the early assembly of the spliceosome. These compounds have different pharmacologic activities and include low (FR901463, FR901464, FR901465, herboxidienes, and pladienolides) and high stability agents (E7107, a pladienolide B-analog, spliceostatin A, and the FR901464 derivative sudemycins). While several agents have only been shown to biologically alter splicing in vitro, a few compounds have also been tested both in vitro and in vivo [56]. For instance, E7107 seems to induce profound inhibition of splicing in cellular models [57], while its effects in humans are still under investigation. More recently, cells carrying SF mutations have been found sensitive to treatment with sulfonamides. Leukemia stem cells showed sensitivity to treatment with the aryl sulfonamides (i.e., indisulam) and this sensitivity correlated with increased DDB1 and CUL4 associated factor 15 (DCAF15) expression levels. Therefore, indisulam [*N*-(3-chloro-7-indolyl)-1,4-benzenedisulfonamide] and other sulfonamides have been investigated for their anticancer activity and demonstrated to drive the recruitment of RNA binding motif protein 39 (RBM39) to DCAF15 for degradation, leading to abnormal mRNA splicing changes (intron retention, exon skipping) [58,59,60]. These observations indicate that cells expressing high levels of DCAF15 might be more susceptible to aryl sulfonamides in general. Another example of the application of small molecules is the anti-proliferative effect of E7070 in combination with idarubicin and cytarabine, which is being investigated in relapsed AML and high-risk MDS (NCT01692197). Moreover, also taking into consideration that protein arginine methyltransferases (PRMTs) inhibitors (MS023, GSK591) were found to influence the growth of *SRSF2* mutant cells, the clinical activity of the PRMT5, GSK3326595, is currently explored in combination with azacitidine in newly-diagnosed MDS and AML (NCT03614728).

### 4.3. Splicing Modulators

As mentioned before, AS is subjected to regulations through post-translational modification. For instance, the Clk (Cdc2-like kinase) family of proteins has been studied because of their role in splicing control. Of note, the benzothiazole compound, TG003, is a very potent inhibitor of the activity of Clk1 [61]. Moreover, this chemical also inhibits the serine/arginine(SR)-rich protein phosphorylation and, for these reasons, it has been proposed for modulation of AS. Additional Clk inhibitors include the small molecules Cpd-1/2/3, which significantly reduce the endogenous phosphorylated SR proteins. Because of the importance of phosphorylation in regulating the activity of SFs, also SRPK1, a phosphokinase that acts on SR proteins and regulates constitutive splicing and AS, may constitute another target for drug development [62].

## 5. Future Perspective

Perturbations in RNA-splicing are present in myeloid malignancies and represent a mechanism influencing cell fate. However, the presence of SF mutations is not an absolute biomarker of splicing abnormalities as they also occur irrespective of SF mutations. These changes, by creating tumor-specific AS phenomena, might represent therapeutic vulnerabilities of cancer, opening new scenarios for targeted approaches [63]. RNA-sequencing technologies have helped to identify a number of pathologic changes in myeloid malignancies and, in the future, will contribute to our understanding of the activity of small molecules targeting the spliceome. More importantly, large scanning genomic technologies have revealed AS to be a mechanism producing a large number of neo-antigens to be investigated for possible immunogenicity. However, if the pre-clinical models show encouraging results, more mature clinical data are needed in order to understand the appropriate context of utilization of AS-targeting agents. Transcriptomic analyses will provide additional information in the future years, especially in regards to the mechanisms beyond the mere presence of non-coding RNAs (ncRNAs), thereby illuminating how they can regulate gene transcription without encoding proteins. Finally, an interesting area of research will be the investigation of circular RNAs (circRNAs), which originate via back-splicing by joining either single or multiple exons or exons with retained introns.

## Data Availability

Not applicable.
